# Endoscopic management of recurrent sump syndrome after surgical choledochoduodenostomy: a stent through two orifices

**DOI:** 10.1055/a-2610-2796

**Published:** 2025-06-26

**Authors:** Jonathan Rozenberg, Reid D. Wasserman, William F. Abel, Paul Yeaton, Patrick Okolo, Vivek Kesar, Varun Kesar

**Affiliations:** 1 Department of Internal Medicine, Virginia Tech Carilion, Roanoke, United States; 2 Department of Internal Medicine, Division of Gastroenterology, Virginia Tech Carilion, Roanoke, United States

A 78-year-old woman presented with recurrent choledocholithiasis. Her medical history included cholecystectomy and surgical choledochoduodenostomy (CDS), and choledocholithiasis consistent with sump syndrome (SS) status post endoscopic retrograde cholangiopancreatography (ERCP) with sphincterotomy, balloon sweep, and placement of a 10 mm × 10 cm fully covered self-expandable metal stent (fcSEMS) into the common bile duct (CBD). She underwent repeat ERCP with removal of the fcSEMs due to stent migration ( Fig. 1 ). Two plastic double-pigtail stents (PDPS; 10 Fr × 6 cm and 7 Fr × 7 cm, respectively) ( Fig. 2 , Fig. 3 ) were placed via a guidewire from the CDS across the papilla ( Fig. 4 ), effectively occluding both sites to prevent further recurrence of sump syndrome. In the 8 months since the procedure, she has remained asymptomatic.

**Fig. 1:**
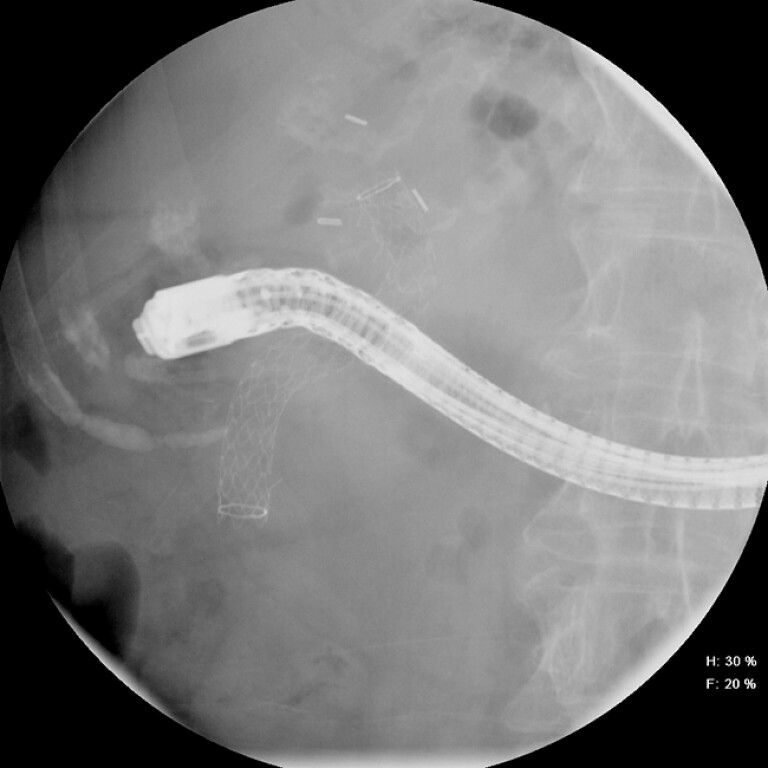
Fluoroscopic image of a fully covered self-expandable metal stent, which migrated from the common bile duct into the choledochoduodenostomy.

**Fig. 2:**
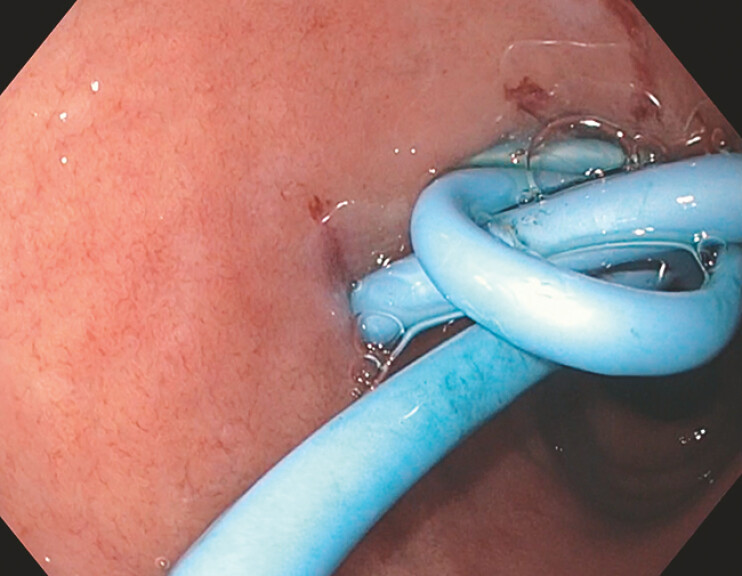
Endoscopic image of a plastic double-pigtail stent placed into the choledochoduodenostomy site.

**Fig. 3:**
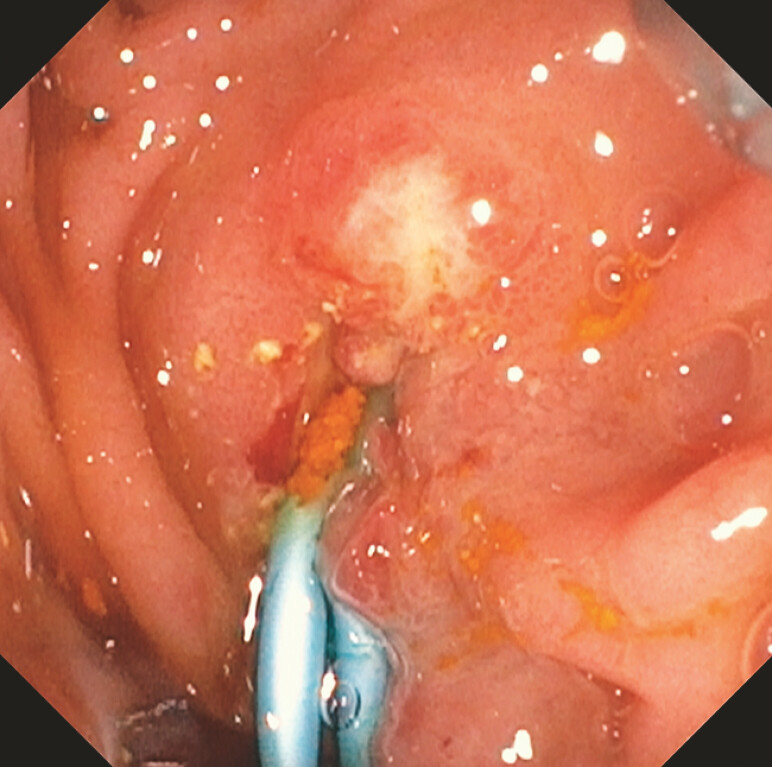
Endoscopic image of a plastic double-pigtail stent placed into the ampulla via the choledochoduodenostomy site.

**Fig. 4:**
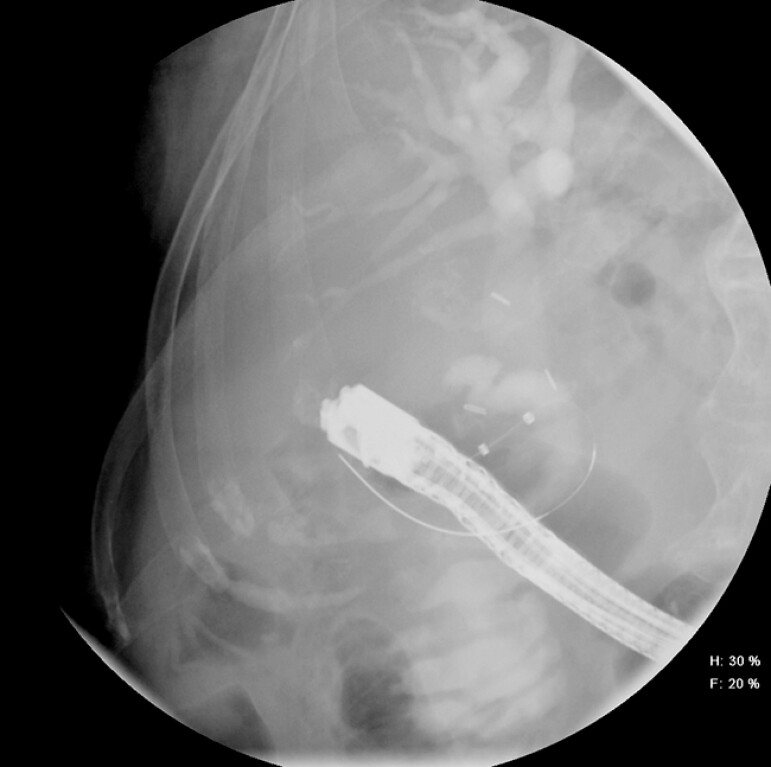
Fluoroscopic image of successful guidewire passage from the choledochoduodenostomy site across the major papilla.

CDS refers to creation of a biliary-enteric anastomosis for the management of biliary stone pathologies and/or malignant biliary obstructions 1 . Consequently, the CBD limb distal to the CDS that extends to the ampulla of Vater, known as the “sump,” can amass stagnant debris, bile, and/or food ( Fig. 5 ), which when in the setting of impaired biliary drainage of the CDS can result in conditions such as ascending cholangitis, otherwise known as sump syndrome 1 2 . ERCP with sphincterotomy and/or debris removal is the mainstay of treatment; however, this does not preclude recurrence of sump syndrome and can necessitate repeat ERCPs and/or further surgical intervention (e.g. Roux-en-Y hepaticojejunostomy with distal CBD resection) 1 2 . The use of PDPS in treating sump syndrome has been documented in case reports wherein choledocholithiasis required the use of lithotripsy followed by PDPS placement into the CDS alone 3 4 . This case depicts successful management and prevention of further recurrence of sump syndrome through placement of two PDPS from a CDS through the major papilla ( Video 1 ).

**Fig. 5:**
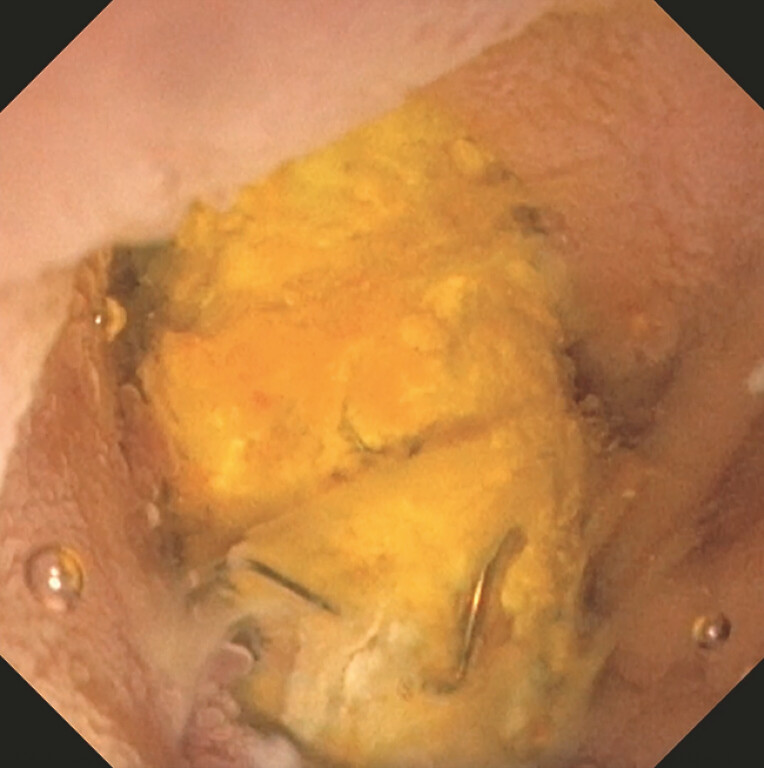
Endoscopic image of a prior fully covered self-expandable metal stent occluded with debris, food obstructing biliary drainage from the choledochoduodenostomy.

Management and further prevention of sump syndrome via the use of two double-pigtail plastic stents at both the choledochoduodenostomy (CDS) and papilla in a patient with a history of surgical CDS with recurrent sump syndrome.Video 1

Endoscopy_UCTN_Code_TTT_1AR_2AK

## Notes

Endoscopy E-Videos https://eref.thieme.de/e-videos
*E-Videos* is an open access online section of the journal *Endoscopy* , reporting on interesting cases and new techniques in gastroenterological endoscopy. All papers include a high-quality video and are published with a Creative Commons CC-BY license. Endoscopy E-Videos qualify for HINARI discounts and waivers and eligibility is automatically checked during the submission process. We grant 100% waivers to articles whose corresponding authors are based in Group A countries and 50% waivers to those who are based in Group B countries as classified by Research4Life (see: https://www.research4life.org/access/eligibility/ ). This section has its own submission website at https://mc.manuscriptcentral.com/e-videos .
